# 

**Published:** 1872-06

**Authors:** 


NEW BOOKS.—Authors and Publishers have been busily engaged during the past winter in getting their works ready for the public, and the result has been some very valuable and interesting works which have already appeared and others which will be ready in a short time.

Messrs Lindsay & Blakiston are about to publish several valuable and instructive works among which we notice a new edition of "Hewitts Diagnosis and Treatment of diseases preculiar to Women, and Clarks outlines of Surgery."

Wm. Wood & Co. announce a new edition of Holmes' System ot Surgery in five volumes, illustrated. They will also shortly publish among others a new edition of Byford's practice of Obstetrics and of Loomis' Physical Diagnosis.

Among the works now going through the press of Messrs Lippincott & Co. we notice a work on Ovarian Tumors by W. L. Atlee, M. D. and Beck's Medical Jurisprudence.

A new edition of Watson's lectures on the Principles and Practice of Physic, and of Gross' System of Surgery, is to be published shortly by Henry C. Lea of Philadelphia. Mr. Lea has also in preparation several other valuable works on medicine and surgery.

Messrs D. Appleton & Co. have several medical works in press, including a treatise on Ovarian Tumors. By E. R. Peaslee, M. D., LL. D. A work on Diseases of the Ovaries. By Dr. T. Spencer Wells. And a Treatise on the Surgical diseases of the male genito-urinary organs, including syphilis. By W. H. Van Buren, A. M., M, D. and Edward L. Keyes, A. M., M. D.

### Books and Pamphlets Received.

Report to the Surgeon General of the United States Army on the minute Anatomy of two cases of Cancer. By Assistant Surgeon J. J. Woodward, U.S. Army Washington D. C. 1872.

Catalogue of the Library of the Surgeon General's office, United States Army. With an alphabetical index of subjects. Supplement to catalogue of Library of Surgeon General's office No. 1. List of American Medical Journals.

Amnesic and Ataxic Aphasia with Agraphia, and temporary right hemaplegia, the result of embolism of the left middle Cerebral Artery. By T. M. B. Cross, M. D. Reprinted from the American practitioner for April 1872.

The Correct Principles of Treatment for Angular Curvature of the Spine. By Benjiman Lee, A. M., M. D. Philadelphia: J. B. Lippincott & Co. 1872. Buffalo: Breed, Lent & Co.

Half-hour Recreations in Popular Science. Dana Estes editor. No. 4. spectrum analysis discoveries. Showing its application in microscopical research, and to discoveries of the physical constitution and movements of the heavenly bodies. From the works of Shellen, Young, Roscoe, Lockyer and others. Boston: Lee & Shepard, 1872. Buffalo: Martin Taylor.

Autumnal Catarrh (Hay Fever) with three maps. By Morrill Wyman, M. D. New York: Hurd & Houghton, 1872. Buffalo: Martin Taylor.

Doctor in Medicine and other papers on Professional subjects. By Stephen Smith M. D. New York: Wm. Wood & Co. 1872.

Minutes of the twenty-third annual meeting of the American Medical Association, held in Philadelphia, May 7th, 8th, 9th, and 10th, 1872. Wm. B. Atkinson, M. D., Secretary 1400 Pine street, Philadelphia, Pa. (Price 50 cents.)

THE BUFFALO

### Medical and Surgical Journal

PUBLISHED MONTHLY,

Containing Original Articles, Reports of Medical Societies and Hospitals, Editorial, Reviews, Correspondence, Army News, etc., etc.; including the usual variety of Medical Periodical Publications. Specimen copies sent on application.

Address Buffalo Medical & Surgical Journal, Buffalo, N. Y.

TERMS OF SUBSCRIPTION, - - $3.00 PER YEAR, IN ADVANCE

